# Transplantation of gut microbiota derived from patients with schizophrenia induces schizophrenia-like behaviors and dysregulated brain transcript response in mice

**DOI:** 10.1038/s41537-024-00460-6

**Published:** 2024-04-08

**Authors:** Nana Wei, Mingliang Ju, Xichen Su, Yan Zhang, Yonghe Huang, Xinyue Rao, Li Cui, Zhibing Lin, Yi Dong

**Affiliations:** 1 Key Laboratory of Adolescent Health Assessment and Exercise Intervention of Ministry of Education, East China Normal University, 200241 Shanghai, China; 2 Shanghai Mental Health Center, Shanghai Jiao Tong University School of Medicine, 200030 Shanghai, China; 3 Shanghai Key Laboratory of Veterinary Biotechnology, School of Agriculture and Biology, Shanghai Jiao Tong University, 200240 Shanghai, China

**Keywords:** Schizophrenia, Microbiology

## Abstract

Schizophrenia (SCZ), as a neurodevelopmental disorder and devastating disease, affects approximately 1% of the world population. Although numerous studies have attempted to elucidate the causes of SCZ occurrence, it is not clearly understood. Recently, the emerging roles of the gut microbiota in a range of brain disorders, including SCZ, have attracted much attention. While the molecular mechanism of gut microbiota in regulating the pathogenesis of SCZ is still lacking. Here, we first confirmed the difference of gut microbiome between SCZ patients and healthy controls, and then, we performed fecal microbiota transplantation (FMT) to clarify the roles of SCZ patients-derived microbiota in a specific pathogen free (SPF) mice model. 16 S rDNA sequencing confirmed that a significant difference of gut microbiome was present between two groups of FMT mice, which has a similar trend with the above human gut microbiome. Furthermore, we found that transplantation of fecal microbiota from SCZ patients into SPF mice was sufficient to induce schizophrenia-like (SCZ-like) symptoms, such as deficits in sociability and hyperactivity. Furthermore, the brains of mice colonized with SCZ microbiota displayed dysregulated transcript response and alternative splicing of SCZ-relevant genes. Moreover, 10 key genes were identified to be correlated with SCZ by an integrative transcriptome data analysis. Finally, 4 key genes were identified to be correlated with the 12 differential genera between two groups of FMT mice. Our results thus demonstrated that the gut microbiome might modify the transcriptomic profile in the brain, thereby modulating social behavior, and our present study can help better understand the link between gut microbiota and SCZ pathogenesis through the gut-brain axis.

## Introduction

Schizophrenia (SCZ) is a chronic and severe neuropsychiatric disease affecting about 1% of the global population^1^, and SCZ causes a heavy burden on public health. Evidence from family-based and twin-base studies supported high heritability in the range of 64–81% in the development of SCZ^2^. Several genome-wide association studies (GWAS) have revealed the presence of associations between genetic variants and SCZ risk^3–5^. The data from GWAS seem to converge on pathways of glutamatergic and dopaminergic systems as well as calcium signaling and immune system genes, and these studies also provide insights into the pathophysiology of SCZ^6^. Although, these data from GWAS have identified risk genes that can explain only a small portion of heritability in SCZ, others such as rare variants and gene-gene interactions are thought to be the additional genetic factors. Besides, other factors such as environmental factors and their interactions with genetic variants may elucidate the causal mechanisms leading to disease phenotypes. Furthermore, the current treatments for SCZ mainly relieve the positive symptoms, but with lesser effectiveness on negative symptoms^7^. Consequently, seeking to identify the role of other non-human genetic factors in the onset of SCZ has become important to investigate. Elucidating the etiology and pathological mechanism of SCZ, looking for novel drug target have valuable applications, and also have been a critical priority in SCZ research.

Gut microbiota, regarded as an exteriorized organ, plays crucial roles in human health^8,9^. Recent studies also have confirmed that the gut microbiota can regulate brain function and host behaviors^10,11^. Although several preliminary clinical studies reported the alternation of gut microbial composition and diversity was associated with SCZ^12–14^, the role of the gut microbiome contributing to SCZ pathogenesis remains unclear. Previous studies showed both the first-episode drug-naïve patients (FSCZ) and medicated patients with SCZ (TSCZ) had an altered microbiome, and emerging clinical and preclinical studies indicate potential relationships between a dysbiosis gut microbiome and SCZ^13–15^, while FSCZ and TSCZ had a distinct changes in gut microbial composition^15^. In animal studies, gut microbiome transfer from medicated patients with SCZ to germ-free (GF) mice and microbiome transfer from drug-free patients with SCZ to SPF mice induced distinct SCZ-like behavioral phenotype^13,14^. Study reported GF mice may be unsuitable model to study the effects of microbiota on brain and behavior due to their permanent neurodevelopmental deficits^16^. A big challenge of SCZ research is the inaccessibility of human materials, especially the brain tissues. Although research on SCZ has not been lacking in recent decades, a suitable SPF animal model for SCZ is still limited.

It is well-known that gut microbiota regulates hypothalamus–pituitary–adrenal (HPA) axis, and the altered HPA axis process is showed be related to the neurodegenerative process in SCZ^17^. Although the correlation between gut microbiota and numerous metabolism pathways were studied, including the metabolism of noradrenaline, dopamine, and serotonin, and the glutamate-glutamine-GABA cycle and kynurenine metabolism^13,14^. Previous studies mainly focus on evaluating the alternations of metabolisms in SCZ pathogenesis and progression. Growing evidence suggests that epigenetic modification plays crucial roles in normal biological process and mental diseases by influencing gene expression transcription, and splicing. Modulation of gene expression, transcription, splicing may be another way of impacting CNS development^5,6,18^. It is necessary to identify the key molecular target linked to the microbiota and SCZ.

In this study, we first made a comparison between the gut microbial communities of antipsychotic-treated patients with SCZ (SCZF group) and healthy control (HCF group) using 16 S rRNA gene sequencing. Then, we performed FMT from antipsychotic-treated patients with SCZ into SPF mice, in parallel with recently published work using microbiome from drug-free patients^14^. Our results provided further evidence that the SCZ patients with antipsychotic treatment have altered microbiome, and the altered gut microbiome can induce SCZ-like symptoms in SPF mice. Our analysis on RNA-sequencing and bioinformatic analysis revealed that Neuroactive ligand-receptor interaction, immune and inflammatory signaling pathways may be related to microbiota-induced SCZ-like behavioral abnormalities in mice. Further, a combined analysis using our data and previously published human brain sequencing data in SCZ further elucidates four genes, including Cybb, Gabre, Baiap3, and Magel2 may be potential molecular targets underlying SCZ-like behaviors.

## Materials and methods

### Human subjects

The present study protocol was conducted in accordance with the Declaration of Helsinki and approved by the Ethics Committee of Shanghai Mental Health Center affiliated to Shanghai Jiao Tong University School of Medicine. All participants provided a written informed consent before any experiments were performed. In this study, 20 SCZ and 15 HC subjects were all from the Shanghai Mental Health Center affiliated to Shanghai Jiao Tong University School of Medicine. All the patients were taking antipsychotics (risperidone, olanzapine, aripiprazole) at the time of recruitment. In addition, only the subjects without any gastrointestinal symptoms were include before sampling. Normal and fresh stool samples were collected and immediately frozen at −80 °C until further use. Detailed information of all participants was presented in Table 1.

**Table 1:** Detailed information of samples tested in this study.

Cohort	Gender	Average age (years)	Therapy	Sample name
Patients with SCZ (SCZF)	Male (*n* = 15)	38.7	Combination	49a-68a
	Female (*n* = 5)			
Healthy controls (HCF)	Male (*n* = 11)	40.5	–	1a-15a
	Female (*n* = 4)			

### DNA extraction and 16S rRNA gene sequencing

Microbial DNA was extracted from fecal samples using TIANamp Stool DNA Kit (TIANGEN, Shanghai, China). The bacteria 16 S rRNA gene (hypervariable V3-V4 region) were amplified by PCR using universal 16 S primers (341F/806 R). PCR was carried out using a Bio-Rad thermal cycler Model C1000 (Bio-Rad, Richmond, CA, USA). Three replicates for each sample were performed, and PCR products were purified with Vazyme VAHTSTM DNA Clean Beads (Vazyme Biotech Co., Ltd., China), and sequenced on an Illumina MiSeq platform by Shanghai Personal Biotechnology Co., Ltd (Shanghai, China) and Magigene Biotechnology Co., Ltd. (Guangzhou, China).

Raw sequences were quality filtered, denoised, merged, and chimera filtered using the DADA2 plugin with DADA2 pipeline^19^. Rarefaction analysis based on the number of sequences and ASVs for each sample was used to evaluate the adequacy of sequencing depth. Alpha diversity was assessed using the diversity index (Shannon, Simpson and Faith’s Phylogenetic Diversity), richness index (Observed species and Chao1) and evenness (Pielou’s evenness)^20^. The Alpha diversity were compared between groups by Kruskall Wallis and dunn’test. Beta diversity was analyzed using the unweighted_UniFrac distances^21^. The differentially abundant taxa between two groups were identified using Linear discriminant analysis (LDA) effect size (LEfSe)^22^. Venn diagram was generated to characterize the common and unique ASVs between groups according to genescloud platform (https://www.genescloud.cn).

### Animals and antibiotic treatment

Male C57BL/6 mice (6 weeks old) were obtained from Shanghai JieSiJie Laboratory Animals Co., Ltd. (Shanghai, China). All animal experiments were approved by the local animal ethics committees (approval number: SV-20201026-02). Mice were randomly grouped and each 4-5 mice were housed in one cage in SPF conditions under a 12 h light/12 h dark cycle. After the acclimation, antibiotic cocktail was prepared to eliminate the initial gut microbiota according to the previous report^23^. Briefly, mice received an oral gavage with 1 mg/kg amphotericin-B for 3 d, and then each mouse was given an antibiotic cocktail of 50 mg/kg vancomycin, 100 mg/kg metronidazole, 100 mg/kg neomycin trisulfate, and 1 mg/kg amphotericin-B for 7 d. Ampicillin was dissolved into drinking water at a concentration 1 g/L for mice. Antibiotic cocktail solution was prepared and freshly used, and all antibiotics were purchased from Sigma-Aldrich (Shanghai, China) and Macklin Biochemical Technology (Shanghai, China).

### FMT experiments

As described previous studies^20,23^, fecal samples from randomly selected five SCZ patients and five demographically matched healthy controls were prepared for FMT. Briefly, fecal samples were suspended with sterile phosphate buffer solution (PBS), and then filtered through 100 µM strainer. The collected supernatant was centrifuged at 6000×*g* for 15 min. The fecal microbiota was suspended in glycerin-PBS and used for transplantation^24^. After receiving antibiotic cocktails for 3 weeks, each mouse received an oral gavage of a volume of 200 μL microbiota suspension for twice each week for continuous 3 weeks.

### Behavioral tests

#### Open-field test

The Open-field test (OFT; reflect spontaneous activity, excitability, and anxiety-like behaviors) was performed in behavior test box of 27.5 cm × 27.5 cm and allowed to explore freely. The movement of each mouse was recorded for 5 min by video camera, and the total travel distance, average speed and time were evaluated in this study.

#### Elevated plus maze test

The elevated plus maze (EPM; reflect anxiety-like behaviors) test was performed in a device, which has two open arms and two closed arms according to the previous study with little modifications^25^. An EPM of four arms with a length of 29 cm and a width of 8 cm. The maze was 17 cm high to the walls. Each mouse was put into the cross section and free access to active for 5 min. Less time and less entering open arms represent the increased of anxiety. The time of activity in open arm and the total entries into open arms were recorded in this study.

#### Forced swimming test

The Forced swimming test (reflect depression and anhedonia-like behavior) was carried out according as described by Porsolt et al. ^26^, with some modifications. The mice were placed into a container with a diameter of 18 cm and a height of 25 cm. Test sessions lasted for 5 min. Mouse was considered to be immobile when it remained floating motionless, only making small movements to keep its head above the water. The immobility time for each mouse was recorded and analyzed in our present study.

### Social interaction behaviors

In the sociability test (reflect sociability, social novelty, and social memory), each mouse was located in a three-chambered box with openings between the chambers. The Three-Chamber Sociability Test (TCST) was composed of three sequential trials: trail 1, the mice were allowed to explore chambers for 5 min; trail 2, social ability (section I, a strange mouse-mouse 1, was placed into a chamber and the test mouse was allowed to explore in the three chambers for 10 min, the time of the direct contact between the strange mouse and empty cage was recorded). trail 3, social novelty preference and social memory (section II, another strange mouse-mouse 2, was placed into the cage in the chamber opposite the mouse 1 from the section I, the time of the direct contact of the test mouse with new strange mouse and previous strange mouse was recorded).

### RNA-seq library preparation and sequencing

Total RNA was extracted from mice whole-brain samples of different groups. Library construction and sequencing were performed using a VAHTS mRNA-seq V3 Library Prep Kit (Illumina, San Diego, CA, USA). The quality was accessed using NanoDrop Spectrophotometer (Thermo Fisher Scientific, MA, USA). RNA sequencing was done on Illumina novaqseq pe150. The data were processed and analyzed using HISAT2 software, DESeq package, topGO and clusterProfiler. DEGs were determined using a cutoff of *p* < 0.05 and |log2FoldChange | > 1. KEGG and GO analyses for DEGs were carried out using topGO and clusterProfiler.

### RNA extraction and quantitative real-time PCR experiments

Total RNA was extracted from the whole brain of different groups as described above, and cDNA was synthesized using cDNA Synthesis Kit (Takara, Dalian, China). The quantitative real-time PCR (qPCR) was performed according to our previous study. Briefly, PCR mixture of 20 μL containing 10 μL SYBR qPCR Master Mix (Vazyme, Nanjing, China), 2 μL cDNA template, 1 μL forward primer and 1 μL reverse primer and 6 μL water. And PCR reaction was performed for 30 s at 95°C, followed by 40 cycles at 95 °C for 10 s, 60 °C for 30 s. The data were calculated using the 2^−△△Ct^ method. The primers used are listed in Table S1. All of the qPCR amplifications were performed in triplicate and were repeated three times.

### Statistical analysis

The significant differences between two groups were analyzed using Student’s *t* test. GraphPad Prism 8 (GraphPad Software, CA, USA) and Origin 2019b software (OriginLab, MA, USA) were used to create common chart and perform common data analysis. A *p* value < 0.05 was considered significant.

## Results

### Characteristics of cases and controls

A total of 20 patients with SCZ and 15 HCs were recruited for this study. Among these patients, 75% were male, and 25% were female. Whereas 80% were male, and 20% were female in case of controls. The mean ages of patients were 38.7, compared with 39.5 for the control subjects. No statistically significant differences were found in the case of age, gender, and body mass index (BMI) between the cases and controls (*p* > 0.05). All the SCZ patients were undergoing an antipsychotic treatment, but they had not taken any antibiotics or probiotics within 1 month before sampling.

### Patients with SCZ exhibit lower gut bacterial diversity

**Fig. 1:**
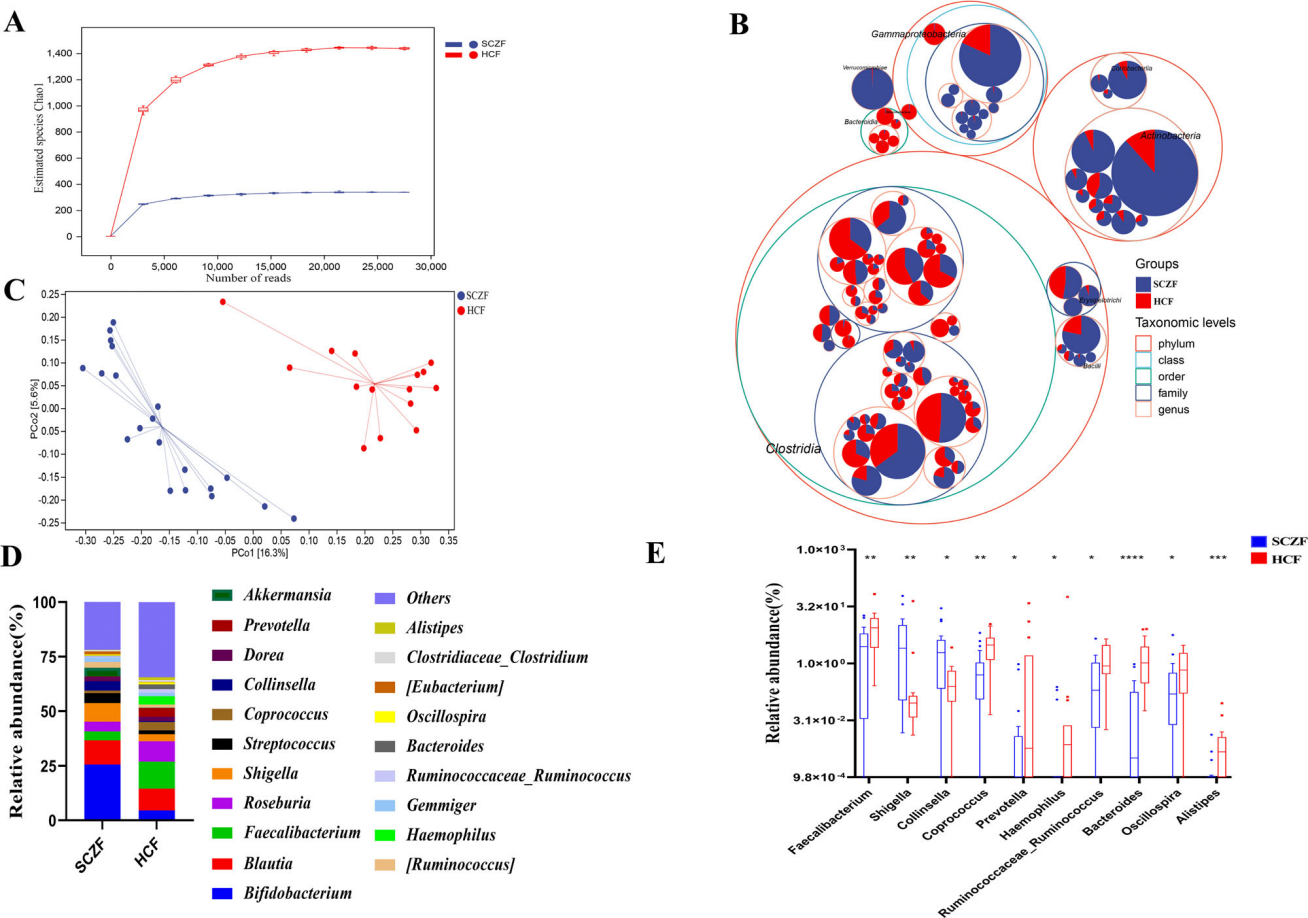
Gut bacterial microbial diversity of SCZ patients and healthy controls. **A** Rarefaction curve comparing SCZF (*n* = 20) and HCF (*n* = 15). **B** Taxonomic differences were based on 16S rRNA sequencing. Taxonomic composition was visualized by circular packing. The largest circles represent phylum, and the inner circles represent class, family, and genus, respectively. The circle sizes represent the mean relative abundance of the taxa. The taxa were colored by sample groups (red for HCF and blue for SCF), whereas the area of the group corresponded to the mean relative abundance of the taxa in each group. **C** PCoA based on unweighted UniFrac measures. **D** Average compositions and relative abundances of the bacterial community in both groups at the genus level. **E** Analysis of differences at the genus level between SCZF and HCF. SCZF patients with SCZ, HCF healthy controls. *p < 0.05, **p < 0.01, ***p < 0.001.

On average, we obtained 43572 high-quality non-singleton reads/sample. Rarefaction analysis of the samples showed that ASV richness in each group approached saturation (Fig. 1A), and it was significantly decreased in the SCZF group versus HCF group. As estimated by six different indexes, gut microbial diversity was significantly decreased in the SCZF versus HCF (*p* < 0.001; Fig. S1A and Table S2). Taxonomic tree in packed circles illustrating the overall abundance of taxa at different levels, revealed distinct differences in the abundance and distribution of taxa between SCZF and HCF (Fig. 1B). Principal coordinate analysis (PCoA) analysis was performed to display the beta diversity, and the results showed a significant distinction of gut microbial communities between both groups (Fig. 1C). A Venn diagram revealed that 774 ASVs were shared between both groups, 2860 ASVs were sole to SCZF group (Fig. S1B). These findings suggested that the gut microbial community of patients with SCZ was characterized by lower diversity.

### Patients with SCZ characterize gut microbiota dysbiosis

We further compared the bacterial composition and abundance of the gut bacterial microbiome between SCZF and HCF. We found that the phyla Firmicutes, Actinobacteria and Proteobacteria together accounted for 93.4% of sequences on average and were the three leading bacteria both in the SCZF and HCF (Fig. S1C). At the phylum level, Firmicutes, Bacteroidetes and Tenericutes were significantly decreased, while Actinobacteria and Proteobacteria were significantly increased in the SCZF, compared with HCF (Fig. S1D and Table S3). At the genus level, *Bifidobacterium*, *Blautia* and *Shigella* were mainly dominated in gut microbiome of patients with SCZ, while *Faecalibacterium*, *Blautia* and *Roseburia* were mainly dominated in gut microbiome of healthy control (Fig. 1D). Compared with HCF, only two genera including *Shigella* and *Collinsella* were increased, while eight genera including *Faecalibacterium*, *Coprococcus*, *Prevotella*, *Haemophilus*, *Ruminococcaceae_Ruminococcus*, *Bacteroide*s, *Oscillospira* and *Alistipes* were depleted in SCZF (all *p* < 0.05; Fig. 1E, Table S4). Among them, *Faecalibacterium*, *Coprococcus* and *Bacteroide*s are essential for producing short-chain fatty acids (SCFAs), which exhibit important anti-inflammatory effects in the host’s inflammatory response^27^. Moreover, it has been reported that *Collinsella* and *Escherichia-Shigella* mainly exhibit pro-inflammatory effects in various disease^28^. Therefore, the decrease in SCFAs-producing bacteria and the increase of pro-inflammatory bacteria may be involved in the inflammatory response in patients with SCZ.

**Fig. 2:**
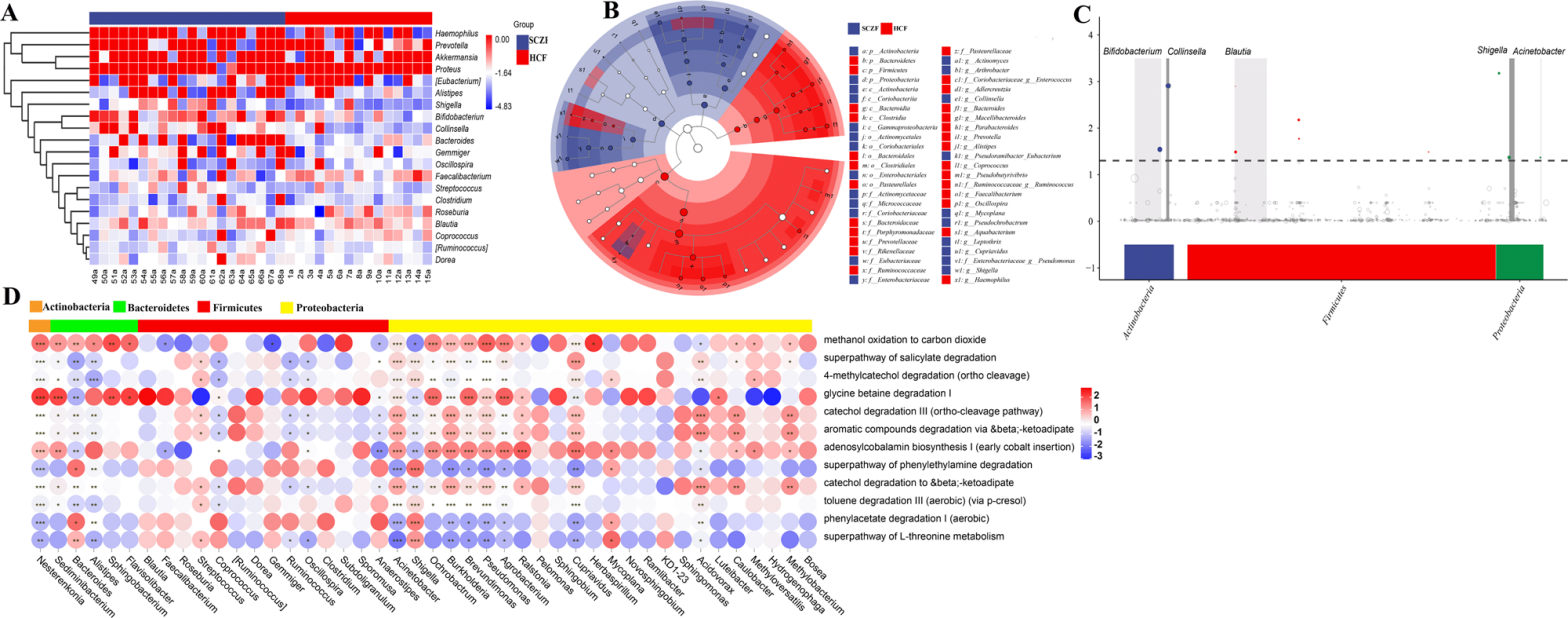
The difference and characteristics of bacteria in different groups. **A** The species composition heat map was created according to the Euclidean distance. **B** LEfSe comparison of gut microbiota between SCZF and HCF groups. Taxonomic cladogram derived from LEfSe analysis of 16S sequences based on the homogeneous ASV_table using the Wilcoxon rank sum test in the R software with correction through FDR. **C** Manhattan plot. The *x*-axis represents the microbial ASV taxonomy at phylum level, and the *y*-axis represents −log10 (adj *p* value). Dots and hollow dots indicate ASVs with and without significant difference, respectively. The color of each marker represents the different taxonomic affiliation of the ASVs. **D** Correlation analysis between metabolism pathway and gut microbes. *p < 0.05, **p < 0.01, ***p < 0.001.

The hierarchical clustering of these changes in taxon abundance demonstrated distinct clusters of in patients with SCZ versus health subjects (Fig. 2A). To determine the variations in gut microbiota composition and specific taxonomic biomarkers between the SCZF and HCF groups, we further performed LEfSe and metagenomeSeq analysis. LEfSe analysis showed that the relative abundance of bacterial taxa between the two groups had significant difference (Fig. 2B). MetagenomeSeq analysis showed that about 10 ASVs were significantly enriched in SCZF, and these ASVs mainly belong to *Bifidobacterium*, *Collinsella*, *Blautia*, *Shigella*, and *Acinetobacter* at the genus level (Fig. 2C). Furthermore, we analyzed the correlation of predicted differential metabolism pathway and gut bacteria, and results showed that twelve metabolism pathways were significantly associated with the relative abundances *Nesterenkonia*, *Bacteroides*, *Acinetobacter*, *Shigella*, *Burkholderia*, *Brevundimonas*, *Pseudomonas*, *Agrobacterium*, *Cupriavidus*, and *Acidovorax* (Fig. 2D).

### Fecal transplant from patients with SCZ induces SCZ-like behaviors in recipient mice

**Fig. 3:**
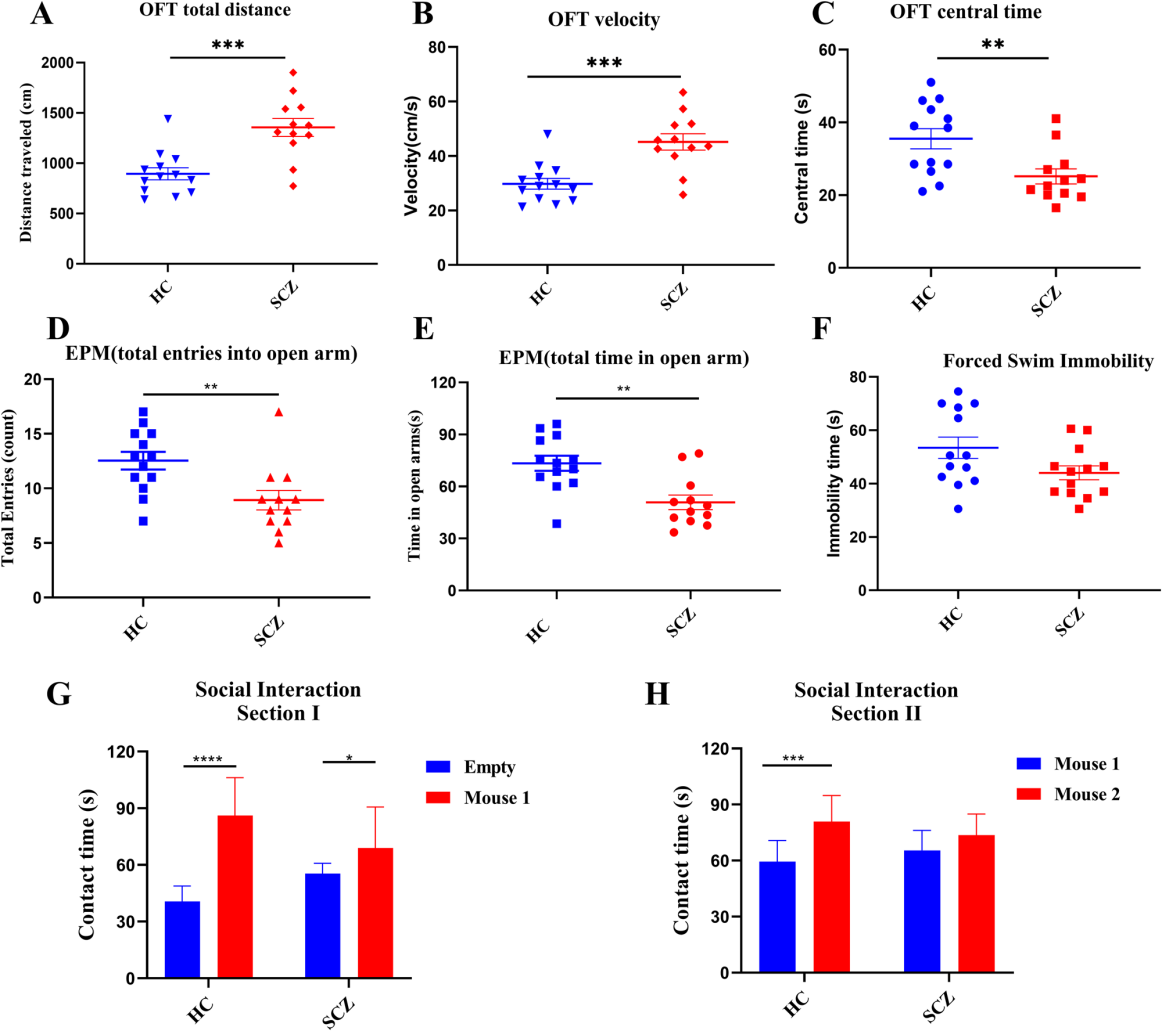
Fecal transplantation from SCZ patients leads to SCZ-like behaviors in mice. **A**–**C** Open field test. Compared to HC fecal microbiota-recipient group, both the total distance traveled (**A**) and average speed (**B**) were significantly increased, but time spent in the center area (**C**) was decreased in SCZ fecal microbiota-recipient mice. **D**, **E** Elevated plus maze test. Compared to the control group, both the number of entries (**D**) and time spent in the open arms (**E**) were significantly reduced in SCZ fecal microbiota-recipient mice. **F** Immobility time showed no significant difference between two groups. **G**, **H** Three-chamber social test. Session I: Different from HC-fecal microbiota-recipient mice group, SCZ fecal microbiota-recipient mice failed to exhibit preference for sociability (**G**) and social novelty (**H**) (HC, *n* = 13; SCZ, *n* = 12). **p < 0.01, ***p < 0.001. Error bars stand for the mean ± SD.

To determine whether SCZ-like behavioral symptoms might be linked with disturbed gut microbiota, we performed FMT experiments in SPF mouse. In the OFT experiment, SCZ fecal microbiota-recipient mice exhibited hyperkinetic behavior (increased total distance traveled, Fig. 3A; higher average speed, Fig. 3B), and increased anxiety (less time spent in the center area, Fig. 3C). In the EPM test, the number of entries and time spent in the open arms were significantly reduced in SCZ fecal microbiota-recipient mice, compared to the fecal microbiota-recipient mice (Fig. 3D, E), suggesting anxiety-like behavior. While, there was no difference between the two groups in the forced swimming test (Fig. 3F). In the sociability test, HC fecal microbiota-recipient mice spent more time to explore the chamber placing a strange mouse than the empty chamber, whereas this significance of SCZ fecal microbiota-recipient group (*p* < 0.0001) was significantly lower than that in the HC fecal microbiota-recipient group (*p* = 0.0392; Fig. 3G). Similarly, HC fecal microbiota-recipient mice spent more time to explore a novel mouse than a familiar mouse, while there was no difference in the time spent to explore a novel and familiar mouse in SCZ fecal microbiota-recipient group (Fig. 3H). Collectively, these behavioral testing results showed that SCZ-like behaviors, including hyperactivity, increased anxiety, impaired social interaction, and memory deficits, were partially recapitulated in SPF mouse by transplanting gut microbiota from SCZ patients.

### Gut microbiome composition in recipient mice transplanted with SCZ or HC fecal microbiota

**Fig. 4:**
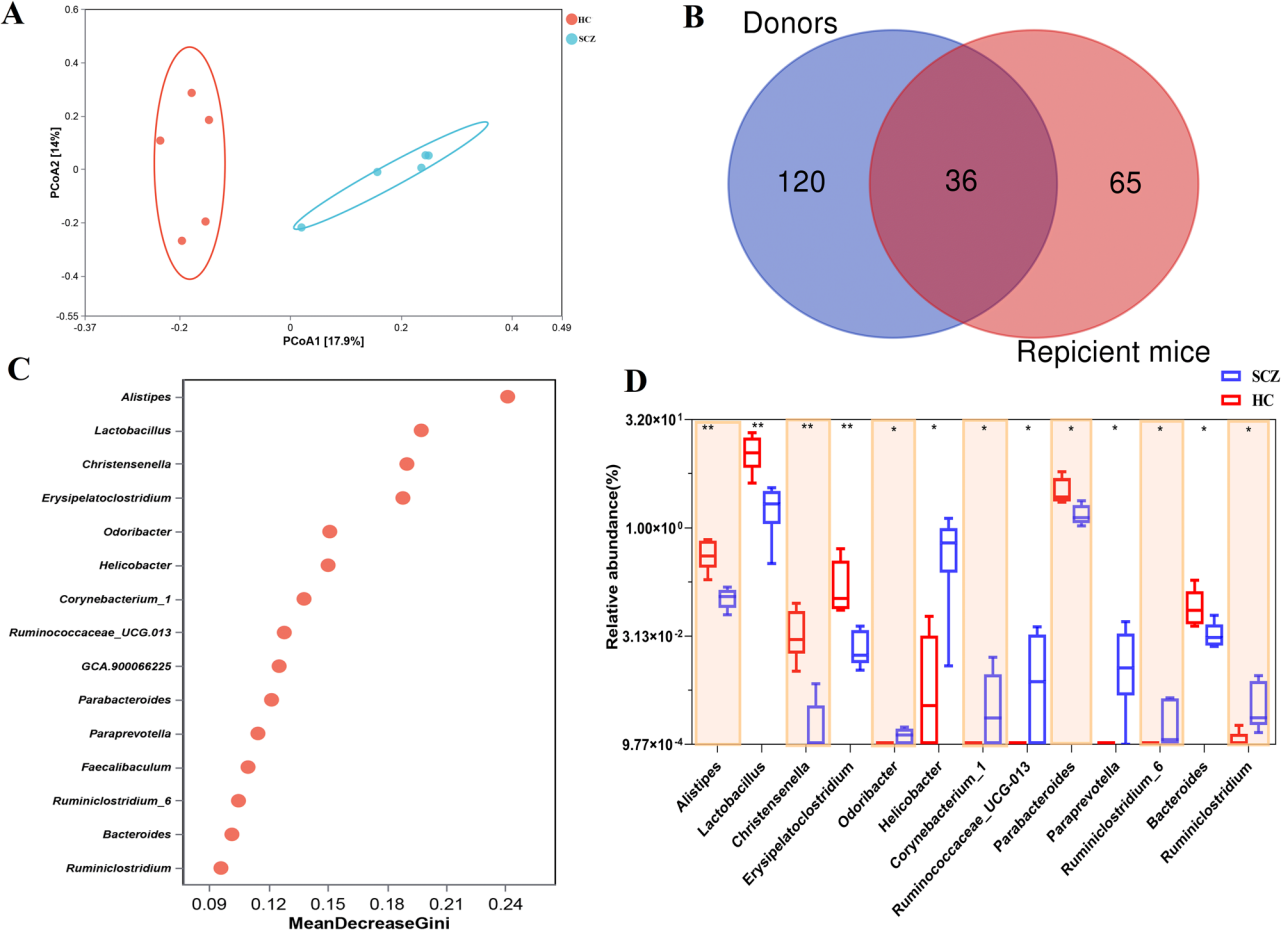
Gut microbial characteristics between SCZ and HC fecal microbiota-recipient mice. **A** PCoA analysis at ASVs level for microbiome of the feces collected from SCZ fecal microbiota-recipient mice (SCZ) and HC mice (HC), based on unweighted_unifrac measures. **B** A Venn diagram displaying the overlaps between human donors and human fecal microbiota-recipient mice. **C** Random forest plot showing the 15 most predictive bacterial genera that differentiate SCZ from HC. **D** Box plots of relative abundance of predictive bacterial genera and the *p* value were calculated using the Mann Whitney test. *p < 0.05, **p < 0.01. Error bars stand for the mean ± SD.

To determine whether the similar gut microbial characteristics of SCZ patients were successfully recurred in the SCZ fecal microbiota-recipient mice, we analyzed the gut microbiota composition of the feces of mice transplanted with SCZ or HC fecal microbiota using 16S rRNA sequencing. The PCoA showed an obvious separation between SCZ and HC fecal microbiota-recipient mice (Fig. 4A). A Venn diagram revealed that 36 of 156 genera presented in donors’ feces were present in the recipient mice gut microbiome (Fig. 4B). To identify the featured gut bacterial microbes that differed between SCZ and HC fecal microbiota-recipient mice, random forest analysis was performed. The 15 bacterial genera with the highest MDA values were shown in Fig. 4C. Among these, seven genera including *Odoribacter*, *Helicobacter*, *Corynebacterium_1*, *Ruminococcaceae_UCG-013*, *Paraprevotella*, and *Ruminiclostridium_6* and *Ruminiclostridium* were more abundant in SCZ fecal microbiota-recipient mice, while the remaining six (*Alistipes*, *Lactobacillus*, *Christensenella*, *Erysipelatoclostridium*, *Parabacteroides*, and *Bacteroides*) were enriched in HC fecal microbiota-recipient mice (Fig. 4D). It is worth mentioning that *Alistipes* showed the similar trend either in SCZ patients or SCZ fecal microbiota-recipient mice, compared to the corresponding controls (Figs.1D and 4D). Previous studies have reported *Alistipes* is involved in various mental diseases including SCZ, by producing SCFA, affecting tryptophan and GABA^27,29,30^. These results further approved that the gut of SPF mice could be re-colonized with the fecal microbiota of SCZ patients or healthy controls by FMT, and *Alistipes* maybe the key genus involved in SCZ.

### Dysregulated transcriptional response in the brain of SCZ fecal microbiota-recipient mice

**Fig. 5:**
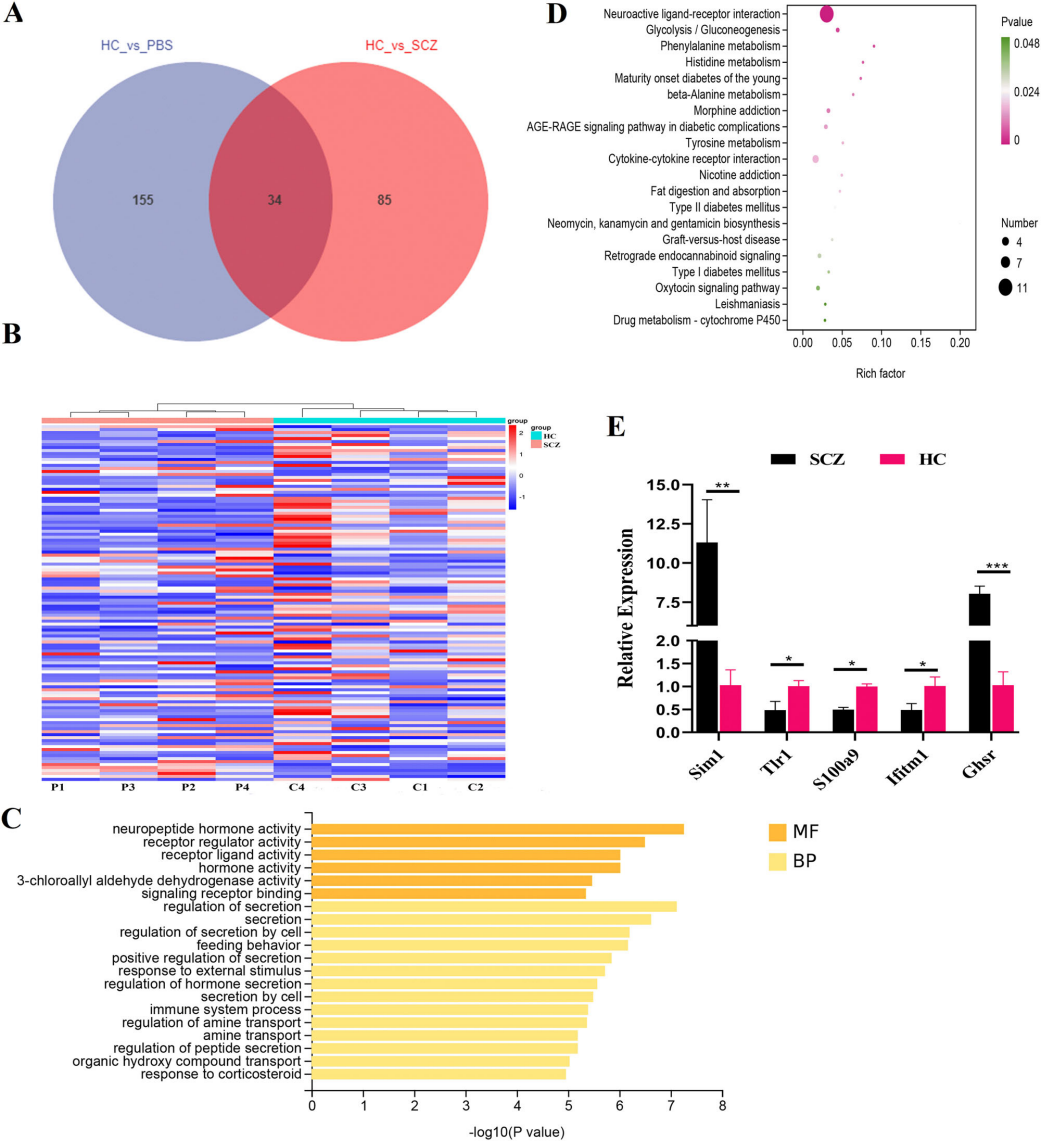
Fecal transplantation from SCZ patients alters the transcriptional response in brain of mice. **A** A Venn diagram displaying the overlaps between different groups. **B** Heatmap displayed hierarchical cluster analysis data for 85 DEGs in the brain of mouse from different groups. Top 20 enriched GO term assignments (**C**) and top 20 KEGG pathways (**D**) for DEGs between two group. **E** Further validation of the RNA-seq data by using qRT-PCR. *p < 0.05, **p < 0.01, ***p < 0.001. Error bars stand for the mean ± SD.

To characterize the molecular mechanisms underlying the behavioral phenotypes in mice transplanted with SCZ fecal microbiota, we performed RNA-seq of the whole brain samples obtained from SCZ and HC fecal microbiota-recipient mice, and PBS was set vehicle control. A Venn diagram revealed that 34 DEGs were overlapped between SCZ vs HC and HS vs PBS groups, while only 85 DEGs were sole to SCZ vs HC group (Fig. 5A). Heat map showed a significant difference of the transcripts between SCZ and HC fecal microbiota-recipient mice group (Fig. 5B). As we expected, FMT from healthy human into SPF mice could also affect mice brain transcript transcriptome (Fig. S2). To rule out this influence factor, only these 85 DEGs were used for GO and KEGG analyses to explore the function of the DEGs between SCZ and HC microbiota-recipient mice group. The top 20 GO terms mainly included neuropeptide hormone activity, receptor regulator and receptor ligand activity, immune system process in brain of SCZ fecal microbiota-recipient mice (Fig. 5C). Bioinformatics analyses suggested that the top 20 enrichment pathways in the recipient mouse brain mainly included Neuroactive ligand-receptor interaction, Phenylalanine metabolism and Histidine metabolism and Cytokine-cytokine receptor interaction (Fig. 5D and Table S5). Furthermore, five DEGs were randomly selected for further validation using qRT-PCR, and the results were consistent with the RNA-seq data (Fig. 5E).

### Overlaps and distinctions of transcriptomes in the brains of SCZ patients and fecal microbiota-recipient mice

**Fig. 6:**
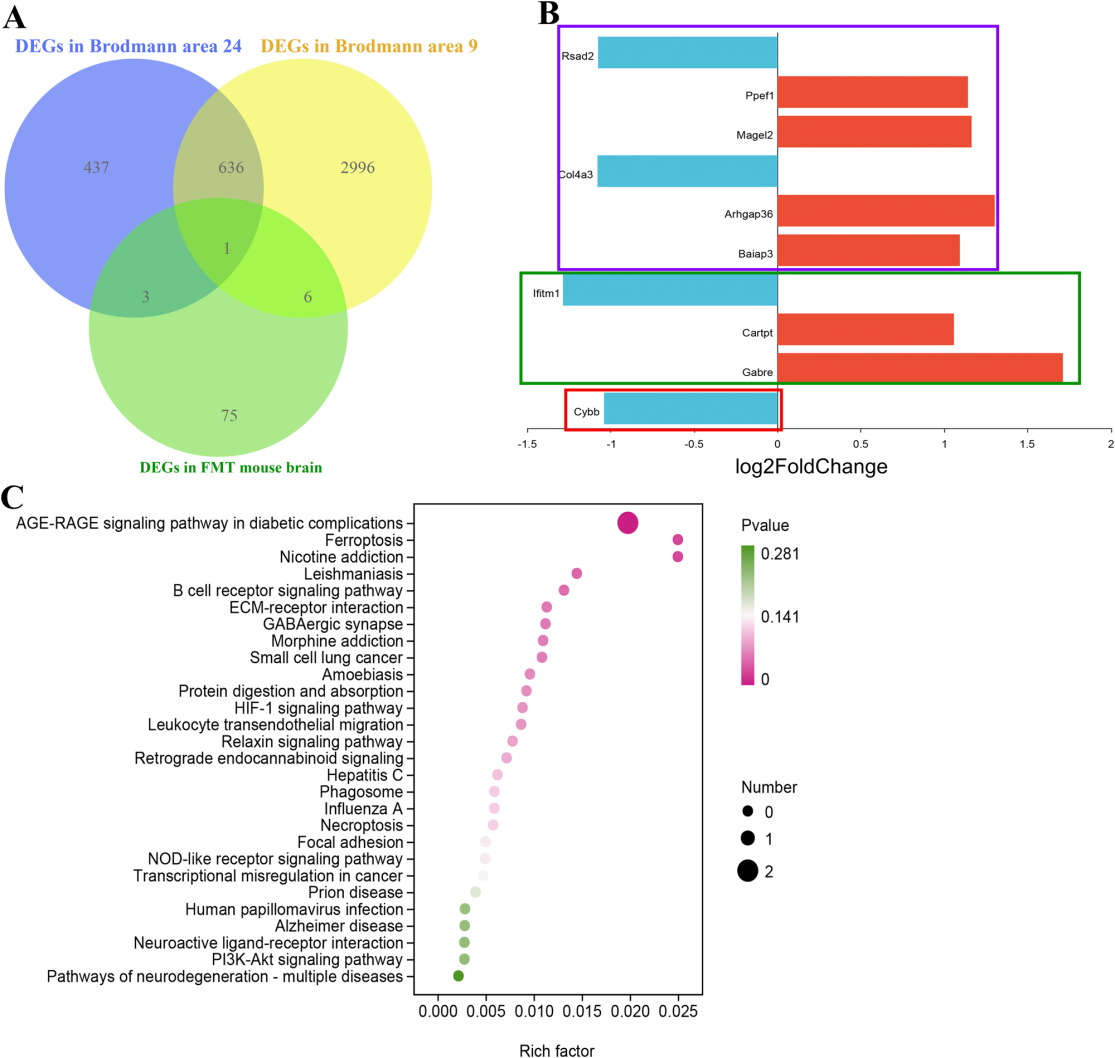
Integrated co-expression of brain transcriptome between SCZ patients and FMT mouse model. Venn diagram (**A**) and bar chart (**B**) showed 4 overlapped DEGs (Genes in the red and green rectangles) between Brodmann area 24 of SCZ patients and fecal microbiota-recipient mice, 7 overlapped DEGs (Genes in the red and purple rectangles) between Brodmann area 9 of SCZ patients and fecal microbiota-recipient mice, and 1 overlapped DEGs (Genes in the red rectangles) both in all the three groups. **C** KEGG modules differentially enriched between samples of brain from SCZ patients and mice model.

To compare transcriptional features and link the association between SCZ patients and FMT mouse model, we further performed bioinformatics analysis to compare the transcriptional dysregulation in the brains of postmortem SCZ patients and fecal microbiota-recipient mice. Venn diagram showed that 7 overlapped DEGs were observed between Brodmann area 9 of SCZ patients and fecal microbiota-recipient mice, and 4 overlapped DEGs were found between Brodmann area 24 of SCZ patients and fecal microbiota-recipient mice (Fig. 6A). While only 1 overlapped DEGs were found to be present among all these three groups (Fig. 6A). All these differential co-expression genes between different two groups included Cybb, Gabre, Cartpt, Ifitm1, Baiap3, Arhgap36, Col4a3, Magel2, Ppef1, and Rsad2 (Fig. 6B and Table S6). The top significantly enriched KEGG pathways were mainly involved in GABAergic synapse, Ferroptosis, HIF-1 signaling pathway, Neuroactive ligand-receptor interaction and Pathways of neurodegeneration-multiple diseases (Fig. 6C).

### Correlation analysis of the enriched or depleted microbes in FMT mice with differential genes in brains

Next, we combined the FMT mouse microbiome with associated brain transcriptomes.

**Fig. 7:**
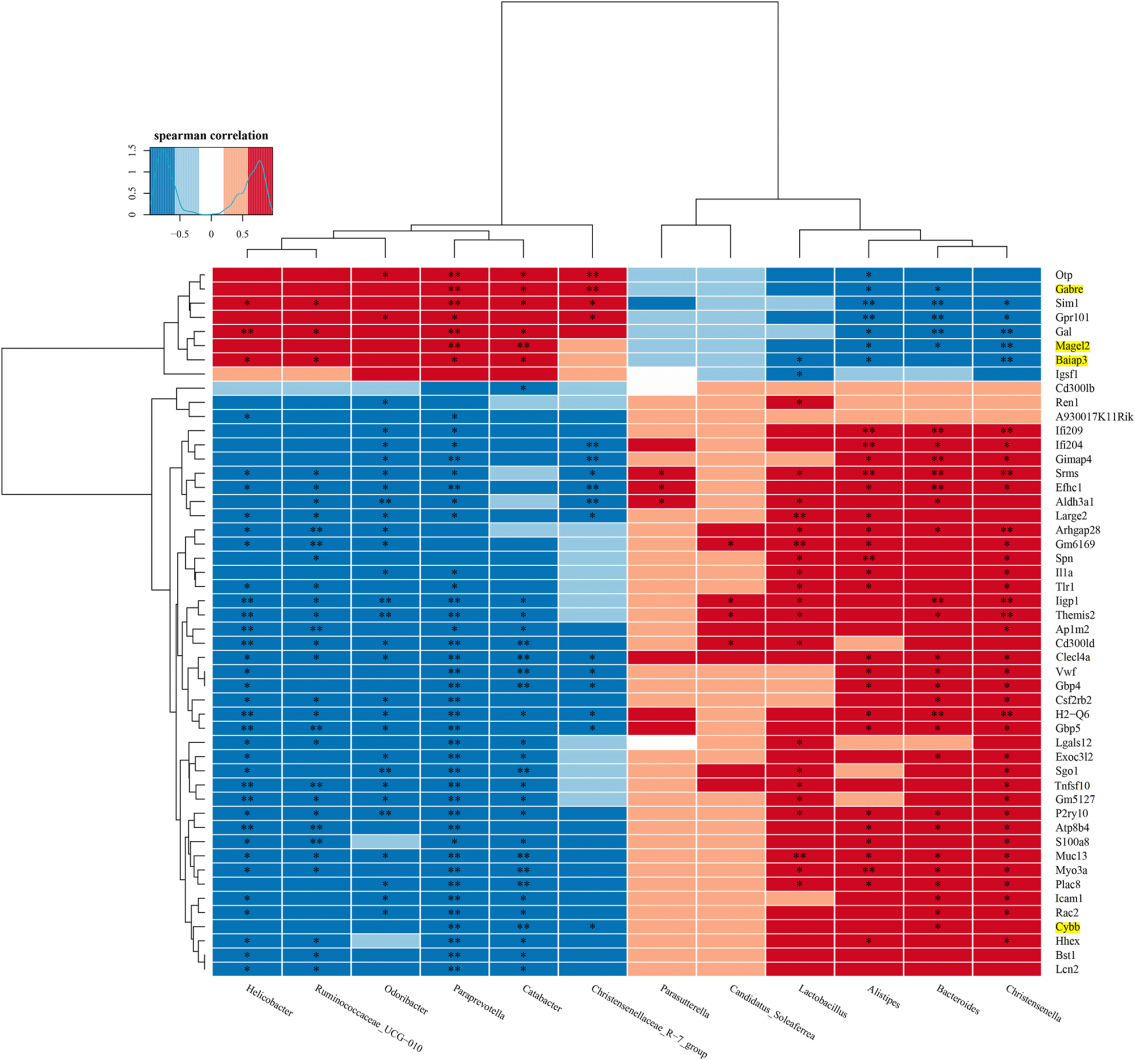
Correlation heatmap of differentially expressed genes and microbiota in the SCZ FMT mouse brain. Yellow background represents the differently expressed overlap genes identified from transcriptomes in the brains of SCZ patients and fecal microbiota-recipient mice. *p < 0.05, **p < 0.01.

A correlation heatmap was constructed to evaluate the covariation between altered gut microbiota genera and altered genes, as shown in Fig. 7. The Spearman’s correlation analysis showed that most of the DEGs were positively correlated with *Lactobacillus*, *Alistipes*, *Bacteroides* and *Christensenell*, and negatively correlated with *Helicobacter*, *Ruminococcaceae_UCG* − *010*, *Odoribacter*, *Paraprevotella* and *Catabacter* (Fig. 7). Notably, four of the above ten overlapped inflammation-related genes (Fig. 6B), including Cybb, Gabre, Baiap3 and Magel2 were identified to be correlated with the 12 differential genera between two groups of FMT mice (Fig. 7). Interestingly, the abundance of *Paraprevotella* and *Catabacter* were all positively correlated with the levels of Gabre, Baiap3 and Magel2, and negatively correlated with the levels of Cybb (*p* < 0.05; Fig. 7). Together, the results suggested that FMT with gut microbiota from SCZ patients could affect gene expression in mice model. Gabre, Baiap3, Magel2, and Cybb maybe potential targets for future research in SCZ.

## Discussion

Mounting evidence links the gut microbiome and brain development and behavior. And, dysbiosed gut microbiota predispose to the onset of various mental disorders, including SCZ, Autism Spectrum Disorder (ASD), and neurodegenerative diseases^31^. Our study found significant alternations in gut microbiota between SCZ patients and HC controls, characterizing a lower microbiota diversity and dysbiosed microbiota composition in patients with SCZ (Fig. 1). Moreover, mice transplanted with fecal microbiota of SCZ patients could induce SCZ-like behavior. Furthermore, we found the mice receiving gut microbiota transfers from patients with SCZ displayed disturbances of transcripts involved in Neuroactive ligand-receptor interaction, inflammation and immunity-related signaling pathways, which has been strongly associated with SCZ pathology. Additionally, integrated sequencing data analysis demonstrated that overlapping DEGs between SCZ patients and fecal microbiota-recipient mice were mainly enriched in GABAergic synapse and inflammation-related signaling pathways. Our results provided further evidence for the key functional roles of gut microbiota and inflammation-related genes in the brain in the pathophysiology of SCZ.

Compared to the HC controls, SCZ patients have a lower microbiome diversity (Fig. 1A), which is consistent with the previous studies^13,32^. Inconsistent microbial community composition and bacteria abundance in SCZ patients and HC controls also exist between our study and the previous study. Our study found that *Shigella* and *Collinsella* were more abundant in SCZ patients, and *Faecalibacterium*, *Coprococcus*, *Prevotella*, *Haemophilus*, *Ruminococcaceae_Ruminococcus*, *Bacteroides,* and *Oscillospira* are significantly abundant in HC controls Fig. 1). While in the Zheng et al.^13^ study, *Megasphera*, *Akkermansia*, *Fusobacterium* and *Prevotella* were significantly more abundant in SCZ patients, and *Citrobacter, Blautia*, *Coprococcus*, *Lachnoclostridium* were more abundant in HC controls. These inconsistencies may be linked to exclusion criteria of potential confounders, such as smoking, antipsychotic treatment, and metabolic illnesses, all of which affect the microbiome. Some subjects with smoking and other metabolic diseases have been excluded in our present study, while the samples with other mental disorders and use of antibiotics, probiotics, and prebiotics within 1 month before sample collection were excluded in Zheng et al.^13^ study.

Recent studies by Zhu et al. and Zheng et al. have reported SCZ-like behaviors in SCZ microbiota-recipient mice by FMT^13,23^. Consistent with our findings, the SCZ microbiota-recipient mice displayed hyperactivity, which is generally regarded to be closely related to positive symptoms of SCZ^33^. Inconsistent behavioral phenotypes in the recipient mice receiving SCZ microbiota also exist between our study and the above two studies. In the TCST, we found SCZ fecal microbiota-recipient mice exhibited impaired social interactions (Fig. 3), whereas Zhu et al. reported similar sociability in SCZ fecal microbiota-recipient mice, compared to the control mice^14^. This inconsistency may be attributable partly to the time of onset in patients with SCZ across these two studies. The onset time of the donors in the Zhu et al. study^14^ was acutely relapsed, while the donors in our study showed chronic symptoms. Impaired sociability in animal models reflects core features of patients with SCZ^34^, our SCZ gut microbiota transplantation mouse model may provide a better understanding of the potential mechanisms underlying SCZ pathophysiology and may lead to novel strategies for developing drug targets of SCZ. Additionally, we found that the GF mouse was used for FMT model in Zheng et al. study, while SPF mouse was used in Zhu et al. study and our study. GF and SPF mice have anatomical and physiological differences, including the immune system, neurodevelopment and behavior^35,36^. An “ideal” mouse model of SCZ may not necessarily exhibit abnormalities in all SCZ-like behaviors. For FMT mouse model in SCZ, the microbiome of SCZ patients and the symptoms of SCZ patients should be considered. Also, different mouse models for FMT, but with the same microbiota origin need to be compared and evaluated in parallel in future studies.

FMT can impact the brain through a variety of different mediators and pathways. Numerous studies have demonstrated that FMT might play an important role in modulating central nervous system (CNS) through the Microbiota-Gut-Brain (MGB) axis, such as changing glycerophospholipid and fatty acyl metabolism, glutamate-glutamine-GABA cycle and kynurenine metabolism^13,14,37^. Other possible mediators of FMT impact on CNS, including altering gene expression, transcription, and splicing, in which FMT may impact CNS development^38,39^. In our present study, four key genes, including Gabre, Baiap3, Magel2 and Cybb, are identified to be potential targets for future research in SCZ. Gabre is mainly expressed in the locus ceruleus, dorsal raphe and cholinergic cells. Gabre is the major inhibitory neurotransmitter receptor responsible for fast inhibition in the basal ganglia. It has been reported that Gaba A receptor is confirmed to be one of the most significant drug targets in the treatment of neuropsychiatric diseases, including epilepsy and anxiety^40^. Baiap3 is mainly expressed in the hypothalamus, central, medial and basomedial amygdaloid nuclei, and the paraventricular nucleus of the thalamus (database: Allen Brain Atlas)^41^. And, these areas play vital roles in modulating autonomic functions and regulating anxiety-related behaviors^42,43^. Magel2 was expressed in arcuate nucleus of hypothalamus, overlapping with vasopressin positive neurons^44^. The abnormality of Magel2 has been confirmed to be involved in neurological disease development^45^. Cybb is mainly expressed in granulocyte, and mouse brain microglia^46^. The deficiency or mutation of Cybb has been reported to be associated with the pathogenesis of chronic granulomatous disease and neurodegenerative disease^47^. In addition, *Paraprevotella* and *Catabacter* were identified to have a strong correlation with the above four genes (Fig. 7). However, future studies clarifying the regulation of these target genes and specific gut bacteria in the pathogenesis of SCZ, are also needed. Recent studies^48,49^ have highlighted the importance of aberrant alternative splicing (AS) of mRNA in neurological diseases, including SCZ. Five AS events were identified including skipping exon (SE), retained intron (RI), alternative 5′ splice site (A5SS), alternative 3′ splice site (A3SS), and mutually exclusive exons (MXE) in our RNA-seq results (data not shown). These findings suggest that FMT not only regulates gene expression in the mouse brain but also affects alternative splicing. Furthermore, AS events of SCZ-like genes will be focused in our further study.

Several limitations to our study must be acknowledged. The sample size in the current study was relatively small. To eliminate interference factors to the maximum extent, some subjects with smoking, other metabolic diseases and gastrointestinal symptoms, have been excluded in this study, and it is difficult to recruit SCZ patients with combination therapy. Thus, only 20 patients with SCZ were included in our present study. However, our results were credible that gut microbiota exerted potential modulation effects on SCZ, which were also consistent with the previous studies^13,14^. Our current results were limited by the fact that microbiota for FMT were combined from all the SCZ patients with different age and sex, and only the whole brain of the recipient mouse was examined. In our future research, a more nuanced approach will be used to examine transcriptomic changes across different brain regions. Despite these limitations, our exploratory results have the potential to better understand the molecular mechanism underlying SCZ pathophysiology, and may lead to new diagnostic and treatment strategies for future SCZ studies.

In summary, our results revealed the effects of gut microbiota on mice by FMT, and our study was also the first time to illustrate the correlation between the microbiota and gene expression levels by 16 s rRNA-seq and brain transcriptome in a SCZ-related FMT mice model. We anticipate our present study can facilitate the understanding of the mechanism underlying SCZ treatment by FMT in clinical practice.

### Supplementary information

Supplenmental Figure (legend) and Supplenmental Tables

## Data Availability

All the raw sequences have been deposited in the NCBI Sequence Read Archive (SRA) under the accession number SRP412965. A STORMS (Strengthening the Organizing and Reporting of Microbiome Studies) checklist is available at Zenodo 10.5281/zenodo.8434471.
