# Supplementary material for: Transplantation of gut microbiota derived from patients with schizophrenia induces schizophrenia-like behaviors and dysregulated brain transcript response in mice
Source: Schizophrenia (Heidelb). 2024 Apr 8;10(1):44. doi: 10.1038/s41537-024-00460-6 (PMC11001608; doi:10.1038/s41537-024-00460-6)
Supplement: Supplementary file 1 — Supplenmental Figure (legend) and Supplenmental Tables [file 41537_2024_460_MOESM1_ESM.docx]

**Supplemental Figure and legends**

**

**

**Figure S1** The characterization and comparison of gut microbial community between SCZF and HCF groups. (A) Alpha diversity (Chao1, Faith_pd, Shannon, Simpson, Pielou_e and Observed_species indexs) measurements distribution of bacterial communities in SCZF and HCF. (B) A Venn diagram displaying the overlaps between groups based on OTU distribution. (C) Average compositions and relative abundance of the bacterial community in SCZF and HCF groups at the phylum level. (D) 3 phylum were significantly decreased, while 2 phylum were significantly increased in SCZF group versus HCF. * *p <* 0.05, ** *p* *<* 0.01, *** *p* *<* 0.001.

**
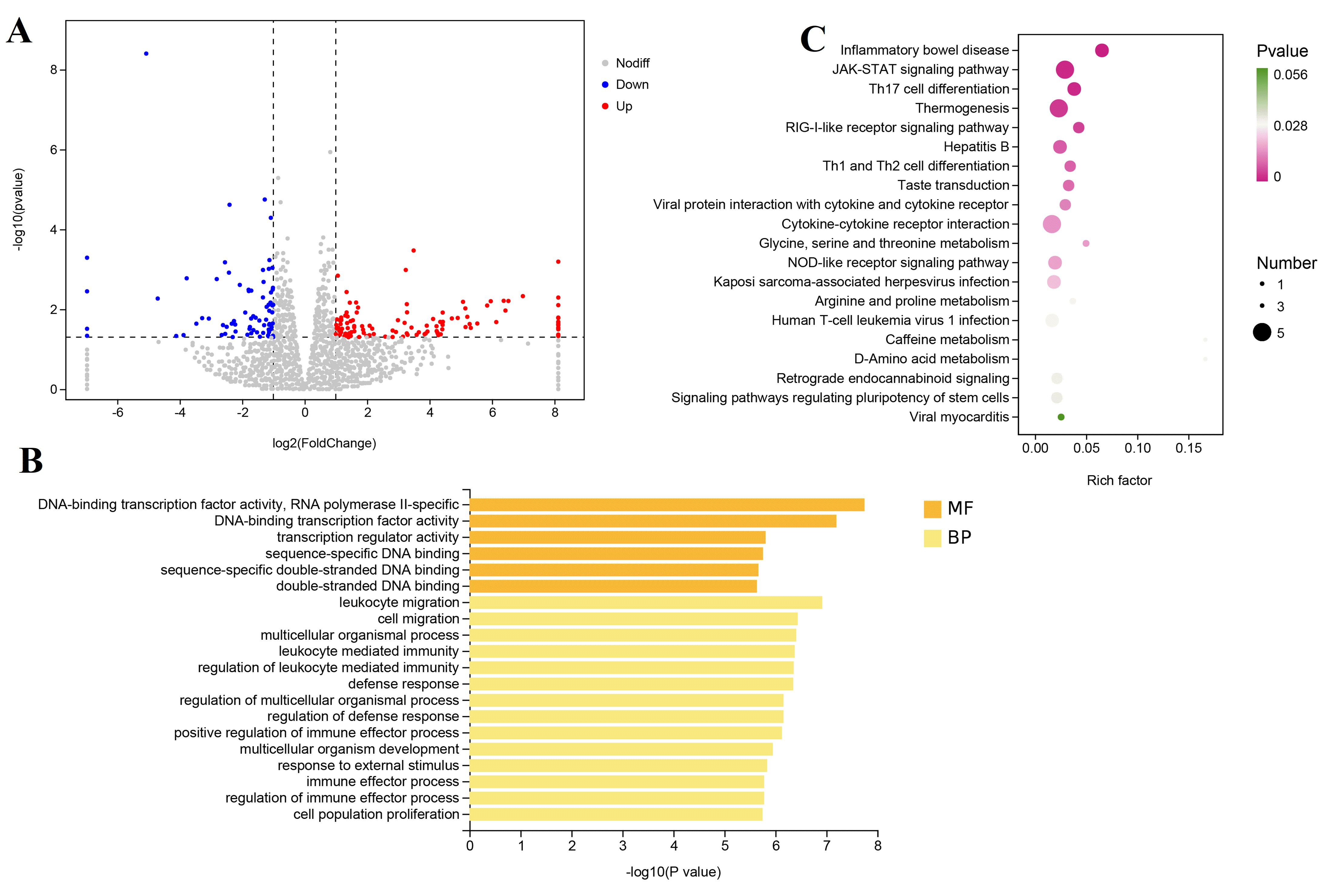
**

**Figure S2 The effects of fecal transplantation from HC controls on transcriptome of mice brain. (A)** Volcano plots of DEGs between the HC fecal microbiota-recipient mice group and PBS vehicle group. Red dots indicate up regulation. Blue dots indicate downregulation. Gray dots indicate no significant difference. **(B)** Heatmap displayed hierarchical cluster analysis data for DEGs in the brain of mouse from different groups. Top 20 enriched GO term assignments **(C)** and top 20 KEGG pathways **(D)** for DEGs between two group.

**Supplemental Tables**

| **Table S1 Quantitative PCR primer sets in this study** | | |
| --- | --- | --- |
| Primer (5'-3') | Gene name | Length of amplification  (bp) |
| F: ACAGGAGTACGAGATCGAACG | Sim1 | 220 |
| R: CTCTGTCACAGCACTCGGAG |  |  |
| F:TGAGGGTCCTGATAATGTCCTAC | Tlr1 | 153 |
| R:AGAGGTCCAAATGCTTGAGGC |  |  |
| F:ATACTCTAGGAAGGAAGGACACC | S100a9 | 129 |
| R:TCCATGATGTCATTTATGAGGGC |  |  |
| F:GACAGCCACCACAATCAACAT | Ifitm1 | 96 |
| R:CCCAGGCAGCAGAAGTTCAT |  |  |
| F:TGGAGATCGCGCAGATCAG | Ghsr | 188 |
| R:CCGGGAACTCTCATCCTTCAG |  |  |

| **Table S2 Alpha-diversity indexes of gut microbiome from different groups** | | | | | | | |
| --- | --- | --- | --- | --- | --- | --- | --- |
| Sample | Chao1 | Faith_pd | Observed_species | Pielou_e | Shannon | Simpson | Group |
| 49a | 247.34 | 27.54 | 232.90 | 0.28 | 2.18 | 0.43 | SCZF |
| 50a | 469.39 | 33.22 | 440.10 | 0.59 | 5.14 | 0.87 |  |
| 51a | 288.42 | 27.03 | 271.60 | 0.58 | 4.66 | 0.84 |  |
| 52a | 264.50 | 24.90 | 246.00 | 0.68 | 5.41 | 0.95 |  |
| 53a | 233.54 | 19.54 | 213.10 | 0.55 | 4.25 | 0.91 |  |
| 54a | 413.89 | 22.61 | 381.70 | 0.60 | 5.11 | 0.91 |  |
| 55a | 802.18 | 43.54 | 772.10 | 0.77 | 7.36 | 0.98 |  |
| 56a | 399.65 | 25.58 | 374.50 | 0.65 | 5.53 | 0.95 |  |
| 57a | 430.23 | 30.83 | 385.40 | 0.50 | 4.33 | 0.86 |  |
| 58a | 244.37 | 17.97 | 228.70 | 0.60 | 4.68 | 0.89 |  |
| 59a | 456.87 | 24.71 | 392.90 | 0.60 | 5.17 | 0.93 |  |
| 60a | 291.71 | 26.35 | 273.50 | 0.62 | 4.99 | 0.92 |  |
| 61a | 199.85 | 20.14 | 179.80 | 0.35 | 2.60 | 0.60 |  |
| 62a | 101.44 | 14.48 | 97.10 | 0.24 | 1.57 | 0.40 |  |
| 63a | 217.31 | 14.60 | 208.20 | 0.69 | 5.28 | 0.96 |  |
| 64a | 318.28 | 19.64 | 295.60 | 0.62 | 5.12 | 0.92 |  |
| 65a | 520.20 | 30.51 | 482.40 | 0.69 | 6.15 | 0.96 |  |
| 66a | 418.98 | 25.96 | 373.00 | 0.69 | 5.87 | 0.96 |  |
| 67a | 150.14 | 12.58 | 127.70 | 0.28 | 1.95 | 0.49 |  |
| 68a | 271.17 | 15.68 | 250.50 | 0.56 | 4.50 | 0.91 |  |
| 1a | 1063.96 | 41.62 | 998.10 | 0.68 | 6.81 | 0.95 | HCF |
| 2a | 1076.48 | 48.97 | 966.40 | 0.64 | 6.39 | 0.95 |  |
| 3a | 1809.66 | 60.57 | 1544.80 | 0.73 | 7.71 | 0.97 |  |
| 4a | 866.06 | 31.34 | 772.90 | 0.68 | 6.53 | 0.95 |  |
| 5a | 1446.64 | 42.48 | 1320.80 | 0.74 | 7.67 | 0.96 |  |
| 6a | 1996.71 | 72.27 | 1783.90 | 0.77 | 8.32 | 0.99 |  |
| 7a | 963.09 | 36.28 | 812.00 | 0.65 | 6.28 | 0.93 |  |
| 8a | 1453.70 | 55.85 | 1268.50 | 0.73 | 7.56 | 0.97 |  |
| 9a | 1699.76 | 55.42 | 1513.50 | 0.77 | 8.18 | 0.98 |  |
| 10a | 1781.52 | 66.37 | 1648.40 | 0.75 | 7.99 | 0.98 |  |
| 11a | 1082.37 | 52.14 | 881.60 | 0.70 | 6.85 | 0.97 |  |
| 12a | 1878.09 | 74.46 | 1757.50 | 0.76 | 8.17 | 0.99 |  |
| 13a | 1626.45 | 48.28 | 1467.40 | 0.78 | 8.19 | 0.99 |  |
| 14a | 1190.71 | 45.24 | 1085.10 | 0.69 | 7.00 | 0.95 |  |
| 15a | 1607.85 | 54.59 | 1515.30 | 0.77 | 8.08 | 0.99 |  |

**Table S3 The different degree of bacterial phylum level (p value) between the SCZF and HCF**

| Genus | SCZF mean | HCF mean | *p* value | Sig_mark |
| --- | --- | --- | --- | --- |
| Firmicutes | 0.49420455 | 0.76850171 | 0.0034 | ** |
| Actinobacteria | 0.30921512 | 0.0558658 | 0.0003 | *** |
| Proteobacteria | 0.14896003 | 0.09141755 | 0.036 | * |
| Bacteroidetes | 0.00517861 | 0.08110717 | <0.0001 | **** |
| Tenericutes | 0.00004267 | 0.00092027 | 0.0324 | * |

**Table S4 The different degree of bacterial genus level (p value) between the SCZF and HCF**

| Genus | SCZF mean | HCF mean | *p* value | Sig_mark |
| --- | --- | --- | --- | --- |
| Faecalibacterium | 0.04100195 | 0.12443785 | 0.0095 | ** |
| Shigella | 0.08461435 | 0.03175074 | 0.0061 | ** |
| Coprococcus | 0.01109485 | 0.03820987 | 0.0159 | * |
| Collinsella | 0.04333916 | 0.00447164 | 0.0016 | ** |
| Prevotella | 0.00087184 | 0.04021616 | 0.0381 | * |
| Haemophilus | 0.00027434 | 3.85E-02 | 0.0444 | * |
| Ruminococcaceae_Ruminococcus | 6.61E-03 | 0.01663821 | 0.0204 | * |
| Bacteroides | 0.00145125 | 0.02128986 | ＜0.0001 | **** |
| Oscillospira | 0.00577838 | 0.00995139 | 0.0435 | * |

**Table S5 Top 20 KEGG terms that are significantly enriched in SCZ fecal microbiota-recipient mice**

| Term description | Pathway ID | | DEGs |
| --- | --- | --- | --- |
| Neuroactive ligand-receptor interaction | | mmu04080 | ENSMUSG00000031340(Gabre\|K05185);ENSMUSG00000024907(Gal\|K05244);ENSMUSG00000020660(Pomc\|K05228);ENSMUSG00000031344(Gabrq\|K05192);ENSMUSG00000023964(Calcr\|K04576);ENSMUSG00000037727(Avp\|K05242);ENSMUSG00000056380(Gpr50\|K04287);ENSMUSG00000005892(Trh\|K05253);ENSMUSG00000027301(Oxt\|K05243);ENSMUSG00000051136(Ghsr\|K04284);ENSMUSG00000044988(Ucn3\|K05257) |
| Cytokine-cytokine receptor interaction | mmu04060 | | ENSMUSG00000096596(Gm10591\|K16062);ENSMUSG00000071714(Csf2rb2\|K04738);ENSMUSG00000027399(Il1a\|K04383);ENSMUSG00000018927(Ccl6\|K05510);ENSMUSG00000068105(Tnfrsf13c\|K05151) |
| AGE-RAGE signaling pathway in diabetic complications | mmu04933 | | ENSMUSG00000015340(Cybb\|K21421);ENSMUSG00000027399(Il1a\|K04383);ENSMUSG00000079465(Col4a3\|K06237) |
| Phenylalanine metabolism | mmu00360 | | ENSMUSG00000075296(Aldh3b2\|K00129);ENSMUSG00000019102(Aldh3a1\|K00129) |
| Glycolysis / Gluconeogenesis | mmu00010 | | ENSMUSG00000041798(Gck\|K12407);ENSMUSG00000075296(Aldh3b2\|K00129);ENSMUSG00000019102(Aldh3a1\|K00129) |
| Leishmaniasis | mmu05140 | | ENSMUSG00000015340(Cybb\|K21421);ENSMUSG00000027399(Il1a\|K04383); |
| Histidine metabolism | mmu00340 | | ENSMUSG00000075296(Aldh3b2\|K00129);ENSMUSG00000019102(Aldh3a1\|K00129) |
| Maturity onset diabetes of the young | mmu04950 | | ENSMUSG00000041798(Gck\|K12407);ENSMUSG00000024986(Hhex\|K08024) |
| beta-Alanine metabolism | mmu00410 | | ENSMUSG00000075296(Aldh3b2\|K00129);ENSMUSG00000019102(Aldh3a1\|K00129) |
| Morphine addiction | mmu05032 | | ENSMUSG00000031340(Gabre\|K05185);ENSMUSG00000032034(Kcnj5\|K04999);ENSMUSG00000031344(Gabrq\|K05192) |
| Tyrosine metabolism | mmu00350 | | ENSMUSG00000075296(Aldh3b2\|K00129);ENSMUSG00000019102(Aldh3a1\|K00129) |
| Nicotine addiction | mmu05033 | | ENSMUSG00000031340(Gabre\|K05185);ENSMUSG00000031344(Gabrq\|K05192) |
| Neomycin, kanamycin and gentamicin biosynthesis | mmu00524 | | ENSMUSG00000041798(Gck\|K12407) |
| Fat digestion and absorption | mmu04975 | | ENSMUSG00000028749(Pla2g2f\|K01047);ENSMUSG00000046008(Pnlip\|K14073) |
| Type II diabetes mellitus | mmu04930 | | ENSMUSG00000054667(Irs4\|K17446);ENSMUSG00000041798(Gck\|K12407) |
| Graft-versus-host disease | mmu05332 | | ENSMUSG00000073409(H2-Q6\|K06751);ENSMUSG00000027399(Il1a\|K04383) |
| Type I diabetes mellitus | mmu04940 | | ENSMUSG00000073409(H2-Q6\|K06751);ENSMUSG00000027399(Il1a\|K04383) |
| Retrograde endocannabinoid signaling | mmu04723 | | ENSMUSG00000031340(Gabre\|K05185);ENSMUSG00000032034(Kcnj5\|K04999);ENSMUSG00000031344(Gabrq\|K05192) |
| Drug metabolism - cytochrome P450 | mmu00982 | | ENSMUSG00000075296(Aldh3b2\|K00129);ENSMUSG00000019102(Aldh3a1\|K00129) |

**Table S6 Overlapped genes between SCZ fecal recipient mice and SCZ patients**

| Genes | in the brain of SCZ fecal recipient mice (up/down) | in Brodmann area 9 of SCZ patients (up/down) | in Brodmann area 24 of SCZ patients (up/down) |
| --- | --- | --- | --- |
| Fgr | down | up | up |
| Clec14a | down | down | down |
| Cybb | down | down | down |
| Tnfsf10 | down | down | down |
| Icam1 | down | up | up |
| Gabre | up | up |  |
| Cartpt | up | up |  |
| Slc38a5 | down | down |  |
| Ifitm1 | down | up |  |
| Baiap3 | up |  | down |
| Arhgap36 | up |  | down |
| Col4a3 | down |  | up |
| Magel2 | up |  | down |
| Ppef1 | up |  | down |
| Rsad2 | down |  | down |
